# Evaluating digital competencies for allied health professionals in the United Kingdom

**DOI:** 10.1177/20552076231176658

**Published:** 2023-05-17

**Authors:** Geraldine Lee, Emma Caton, Annemarie Knight

**Affiliations:** 1 Florence Nightingale Faculty of Nursing, Midwifery and Palliative Care, 4616 King's College London, London, UK; 2 Department of Nutritional Sciences, King's College London, London, UK

**Keywords:** Digital competencies, allied health professionals, digital technology

## Abstract

The Covid-19 pandemic accelerated the move to virtual and remote consultations in clinical practice with digital technologies widely implemented. eHealth interventions and use of applications in a variety of conditions means that patients and their families, as well as healthcare professionals, can access and interpret data in real-time, as well as providing trends in various clinical parameters including blood pressure for instance. Despite the aim of digital transformation in the National Health Service in the United Kingdom, this has not been fully realised and there is no consensus on the skills and competencies required for allied health professionals (AHPs). This qualitative study undertook two focus groups with twelve AHPs to evaluate the AHP Digital Competency Framework in the UK. The participants recognised the importance of a digital technology in their clinical practice and perceived digital literacy as essential for AHPs. In relation to the AHP framework, participants agreed that competencies in digital technology were clinically relevant, and assessment of these competencies should be performed regularly in practice. However, the majority were unaware of the AHP digital competency framework and suggested improvements to optimise its use in practice and identified areas for improvement. Overall, the AHP Digital Competency Framework has the potential, with better dissemination and further refinement of the wording, to become a useful tool to support the enhancement of digital competency in AHPs and improve the delivery of patient care.

## Introduction

Digital technology plays an important role in health and social care practice and can provide significant benefits for patient care. For example, the introduction of care coordination systems, such as the Electronic Health Record, has allowed for vast improvements in the documentation and retrieval of patient information, and remote monitoring devices have enabled healthcare staff to monitor patients and provide support outside of the clinical environment. With the need to reduce or cease face-to-face consultations during Covid-19, virtual and remote consultations were introduced with many healthcare professionals adapting accordingly.^1^ However, despite the unprecedented pace of uptake of digital technologies and these changes remaining in place post-Covid, it has become apparent that little or no digital skills training or education for staff has been implemented.^2^ The full digital transformation of healthcare services has yet to be realised within the UK, and strategies have been detailed in the 2019 National Health Service (NHS) Long Term Plan to further implement and upgrade digitally enabled care across all NHS services.^3^ It is important to consider the skills and competencies healthcare practitioners require.

Allied health professionals (AHPs) are the third largest workforce within the NHS and play a crucial role in improving the health and wellbeing of patients and supporting them to live full and active lives.^4^ Enabling AHPs to use information and technology can help improve AHP services on both a national and local level,^5^ and has been identified by AHPs into Action as one of four key priorities in the transformation of health, care, and wellbeing in England.^6^ A meta-synthesis of competency standards reported a major gap in digital health with a need for specific digital health competencies.^7^

The AHP Digital Competency Framework has been developed as part of a Topol Digital Health Fellowship to support the enhancement of digital competency in AHPs.^8^ The document includes 124 competencies across 10 domains (See Table 1) and is intended for use across all 14 AHPs from Band 3 through to Band 9 (these are the Agenda for Change pay grades within the NHS: Band 3—an emergency care assistant for example to Band 9—chief finance manager).

**Table 1.** Allied health professional competency framework domains.

Domain number	Domain name	Capabilities assessed
1	General	Continued Professional Development. Attitudes and beliefs. Behaviours. Foundational computer skills.
2	Data management & Clinical informatics	Information governance, cyber security and privacy. Interoperability. Data evaluation and analytics. Clinical coding.
3	Records, Assessments & Plans	Structured vs. unstructured data in EHR. Data capture at point of care. EHR system design and modification.
4	Transfer of care	Risks of transferring patient data. Data sharing. Personalised care.
5	Medicines management and optimisation	e-Prescription. Automated dispensing. Independent prescribing. Patient Group Directions/Patient Specific Directions in EHR Electronic medicine optimisation pathways.
6	Orders & Results management	Diagnostic testing and screening. Pathology/laboratory testing and medical imaging. Viewing, sharing and storing data. Associated AI and machine learning.
7	Assets & Resource optimisation	Organisation: Patient administration systems, bed management systems, business intelligence and support. Personal: Electronic staff records, HR systems, e-Rostering.
8	Decision Support	Automated evidence based guidelines with systems. Associated AI and machine learning. Risks and bias Professional responsibility.
9	Digital therapeutics	Clinically assured health information. Remote care pathways. mHealth apps and devices. Other patient facing technologies.
10	Meta-competencies	Leadership skills. Development of strategy to guide digital transformation. Quality improvement and research. Change management. Governance.

Previous research using the AHP Digital Competency Framework has demonstrated that AHPs have moderate–high levels of confidence using digital technology at work and have moderate–high levels of motivation to learn.^9^ Despite this, over half of the competencies (58.8%) were identified as being high priority for improvement, meaning that more than 50% of participants perceived their ability to perform the competency as poor or very poor. Particular areas for improvement included decision support, digital leadership, and the use of the Electronic Health Record.

Another finding of the study was the high rates of inapplicability for some of the competencies included in the Framework. Whilst the document aims to encompass all allied health professions, and the multiple bands within each role, the number of competencies rated ‘not applicable’ may indicate a limitation of the framework in relation to its suitability for use in clinical practice. As a result, the current study aimed to explore the views of AHPs towards the AHP Digital Competency Framework and evaluate its suitability for use within healthcare education and practice.

## Methods

### Design and recruitment

The current study employed a qualitative research design, with the use of focus groups, to explore the opinions of AHPs towards the AHP Digital Competency Framework. The study advert was distributed to AHPs at Guy and St Thomas’ NHS Foundation Trust via email and was promoted on the social media platform Twitter. Participants needed to be employed as an AHP in the UK, and have a valid NHS email address, in order to be eligible for the study. A convenience sample of AHPs was recruited and twelve individuals showed interest and agreed to participate. Informed consent was obtained from all participants prior to the beginning of the focus groups. The study was designed and executed with the consolidated criteria for reporting qualitative research.^10^

### Materials and procedure

Two focus groups were conducted with AHPs in June and July 2022 as part of a larger study that investigated the views of health professionals on AHP Digital Competency Framework. The focus groups were conducted online via MS Teams with each participant located in their workplace and lasted for ∼60 minutes. Two researchers were present during the focus groups; GL who facilitated the discussion, and EC who was responsible for taking notes. GL is an experienced nurse researcher in qualitative methods who also runs a weekly clinic and is a nurse prescriber and is also a subject matter expert on digital technologies and curriculum development. EC has a background in psychology and is a research associate with experience of undertaking focus groups and thematic coding. AMK is a dietitian, educator and active researcher within the area of nutrition and dietetics who has experience with qualitative methods.

Participants were provided with a copy of the AHP Digital Competency Framework prior to the beginning of the focus groups. A topic guide was developed to explore participants’ thoughts and opinions towards the Competency Framework. Following introductions, participants were encouraged to share their general perceptions of digital technology and the need for digital competencies in the workplace. Each domain from the Competency Framework was then presented and discussed within the group. Particular focus was given to the relevancy of each domain to clinical practice, the structure and terminology of the competency statements, and the ways in which the competencies could potentially be assessed. The focus groups were recorded, with permission from participants, and manually transcribed.

### Analysis

A topic guide was developed by GL an experienced researcher in qualitative methods to explore the views of the AHPs staff in relation to digital technology, digital competencies, and the AHP Digital Competency Framework. A copy of the framework was sent to all participants prior to the focus groups with open questions developed by the researchers on evaluating the framework in a methodical manner. Examples of some of the questions used are: (i) ‘*Is the framework clinically relevant to your profession?*’ and (ii) ‘*Could this competency be easily assessed in your practice?*’, and more specifically, participants were asked ‘*What level do you think you are working at within this competency?*’ Internal validity was sought with the focus group held by GL is a nurse academic and programme lead for an advanced practice Masters for AHPs and in terms of reflexivity and reducing possible bias, the researcher had not interacted with any of the participants prior to the interview but with a declared interest in digital health and competencies. The researcher made note of comments which mapped directly onto specific domains of the AHP Digital Competency Framework, to ensure this was accurately reflected in the results. Respondent validation was also used to ensure the responses given were valid for all participants and GL rephrasing some points to ensure validity. The focus group was audio- and video-recorded, with permission from participants, and then manually transcribed. MS Teams has a recording function with generates a transcript and this was used along with manual transcription by EC. The transcript draft and focus group notes were then checked and re-read by EC and checked by a second researcher (GL) for accuracy and then systematically and manually coded by EC and checked by GL. In terms of trustworthiness and rigour, the audio and video recording of the focus groups ensured results were dependable and confirmable with a researcher who is not an AHP undertaking the focus groups (GL) and independent researcher analysing the transcripts (EC). Similar ideas drawn together to form themes and derived from the data using Braun and Clarke's reflexive thematic analysis.^11,12^ Final themes were agreed using a consensus approach. The transcript was then re-visited to ensure that the themes were representative of the ideas and views expressed during the focus group. One round of analysis was undertaken and there were no disagreements on the themes by the three researchers.

### Ethics

The study was registered with the College Research Ethics Committee under the minimal ethical risk process at King's College London prior to recruitment in May 2022 with reference number: MRA21/22-32005.

## Results

A total of 12 AHPs took part in the study (Focus group 1: *n*  =  7; Focus group 2: *n*  =  5) and 11 participants were female. The majority of participants were dietitians (*n*  =  10), with one occupational therapist and one physiotherapist also taking part. All participants were senior clinicians working in the NHS (Band 7, *n*  =  10 and Band 8, *n*  =  2).

### Themes

#### Theme 1: General perceptions of digital technology

The AHPs acknowledged that digital technology plays a significant role within the NHS and has multiple benefits for the healthcare service.

*From a service level side … the ability to work more remotely makes the team more productive … I think there's a way to improve efficiencies as well*. (Participant 11)

On the other hand, participants also highlighted that substantial progress is still needed to establish digital equality across all healthcare users, and to ensure that patients fully understand the implications of sharing their data in the digital age.

*…the newer continuous glucose monitors are only compatible with certain mobile phones, and some of the families who are on low income have to buy a more modern mobile phone to be compatible with the technology that's being provided … there's huge variation out there*. (Participant 9)

*I also think data systems and GDPR information and sharing et cetera is very incomprehensible … if they [patients] don't get it, do they know what they're agreeing? Do they know what's informed and not informed, that they're agreeing to share their data?* (Participant 1)

Participants perceived good digital literacy as ‘essential’ for the AHP role, especially given the growing importance of digital technology on the future of the healthcare services.

*I think it [digital literacy] is essential. It's my view that the digital future is the only way that the NHS can hope to survive.* (Participant 3)

#### Theme 2: Current and future use of competency framework in practice

Only 1 out of 12 participants had heard of the AHP Digital Competency Framework prior to taking part in the focus groups, suggesting that the use of this framework within practice is currently limited. Participants identified several limitations of the framework and suggested that improvements would need to be made before the document could be considered fit-for-purpose.

*As it is at the moment, I wouldn't see any place for using it [the AHP Digital Competency Framework] in my practice. And I wouldn't see any place for using in the practice of people I manage either*. (Participant 3)

Nevertheless, participants explained that it would be useful to use the framework as a criterion against which they could evaluate their current levels of competency and identify areas for improvement.

*It's always good to be able to get that feedback or have a set criteria that you're trying to work towards or work to achieve*. (Participant 6)

*A competency framework would be much more about identifying your challenges, and putting an action plan together to meet them*. (Participant 3)

#### Theme 3: Assessment of digital competencies

There was consensus among participants that digital competencies should be assessed. It was suggested by the AHPs that digital skills should be a mandatory competency that is regularly reviewed within the workplace to ensure that staff have the appropriate skills to fulfil their role.

*if there was a way of being assessed on all the competencies relevant to that role- so if they were designed like you do with mandatory training and things like that, that you are expected to go through.* (Participant 4)

*Maybe it becomes part of competency… like it would be reviewed in a good way, not every year but every few years, just so that we know we've upskilled a whole workforce*. (Participant 1)

Participants suggested that digital competencies should be assessed by other people within the workplace. Some participants felt that peer assessment would be beneficial in receiving an alternative perspective on their levels of competency and identifying areas for improvement.

*You could do a peer-assessment or something like that. I think sometimes it's not until somebody feeds back to you on your own practice that there's certain things that you might notice don't work so well.* (Participant 6)

Nevertheless, some participants considered peer assessment to be too subjective for a formal exercise and were concerned that the assessment could be influenced by the examiners own understanding and interpretation of digital competence.

*I think it gets a bit grey when you do peer assessments … you may have someone who has a very different level of understanding and competency to the next assessor. I think it opens up a quite a large amount of subjectivity on something that should be quite objective*. (Participant 11)

Participants suggested that being assessed by a manager or senior member of staff would be more appropriate than peer assessment and could also help senior management to identify staff who may require additional support, or highlight competencies that staff find challenging so that more education and training can be provided.

*I think probably formal assessments would be the best way to do it … if a colleague assesses them or a senior colleague, and that happens for everyone, then you might get a clear idea of who has weaknesses and might need a little bit more support to get up to speed*. (Participant 9)

In relation to the AHP Digital Competency Framework, participants expressed that they were unsure how the document would be used to actively demonstrate their levels of competence. They highlighted that the majority of competencies began with the words ‘I have’ which may prompt a ‘yes/no’ answer without the need for staff to consider how they meet the competency or complete any action to demonstrate that the competency has been met.

*In some of them I understand what they're trying to say, but then how you would actually demonstrate that, I'd be intrigued to know*. (Participant 6)

*For me, a sentence like that [beginning with ‘I have'] just directs you towards saying yes without actually making any action. I could just read that and say yes.* (Participant 3)

Furthermore, the use of ‘I have’ statements also presented the competencies as binary concepts. Participant noted how levels of ability fluctuate over time and staff may meet the competency to some extent, but still require support to fully develop their understanding of the process.

*It's too vague. It's just, ‘yes I do’ or ‘no I don't’*. (Participant 9)

*I think also some of this is you use it or you lose it. I was really good at SPSS at one time … but if I was presented with [SPSS] today, I would say I'm familiar with it, but I don't know if I'm competent.* (Participant 2)

It was suggested that a task-based element should be included in the framework so that staff are able to reflect on their knowledge and demonstrate that they have the appropriate skills to meet the competency.

*If there could be some way of it [competencies] being task based, where it would say create a spreadsheet, work out the formula, input the formula for this much data. Sometimes in the doing you're learning as well as being assessed*. (Participant 2)

#### Theme 4: Structure and content of digital competencies

Participants felt that many of the statements within the AHP Digital Competency Framework were too long and wordy, making it difficult to understand what was required by the competency. Participants found it overwhelming to read through the document, given the large amount of information presented, and suggested that the competencies should be made shorter and worded in a way that is accessible and easy to understand.

*I'm just reading through number 9 … What does that actually mean? I can't say that in one sentence…* *I don't know whether they're just trying to cram too many things into one little competency, but they don't read that fluently either.* (Participant 11)

Similarly, some AHPs felt that the competencies were too vague and ambiguous. Given the huge variety of different digital systems used within the healthcare service, participants were unsure what platforms or processes were being referred to in the competency statements and mentioned that it would be useful if specific examples were provided.

*Because we use so many different systems, I've never used attend anywhere, but I can use Teams and I can use Zoom. I couldn't use half of the systems that you guys use because I've not been trained, so it's a bit difficult to know if you can do that or not.* (Participant 8)

*I think it comes back to the point that it would be better if each one was just very short and simple, and if there was maybe a specific example that was relatable.* (Participant 9)

A further criticism of the AHP Digital Competency Framework was that not all of the competencies are relevant to the role of an AHP. For example, some competencies described processes which are typically performed by administration staff or those in a managerial role, and beyond the scope of practice for an AHP. Furthermore, some of the competencies replicated information that is already provided in other training programmes or addressed areas of practice that are governed by other departments or professional regulatory bodies (see Table 2).

*Is it trying to be so comprehensive that then you would maybe go through and identify ‘these are the ones that apply to us’, and you cherry-pick? I haven't been able to read it all, but it feels like 50% of it would not apply.* (Participant 2)

*I think we already do the mandatory training about data protection and information governance, don't we? So that's already done really*. (Participant 7)

*I think so often there's repetition within all of these different spheres, and it feels like this is another example of a lot of things being repeated that is already being done*. (Participant 4)

Participants recommended re-structuring the document to include core competencies that are essential for all staff, and additional competencies that relate to specific job roles or Bands.

*I think it's a good point about having a core base of competencies that everybody should reach … going forward everybody's got those core competencies. Then it's only when you go up the Bands that there's add-ons*. (Participant 7)

Participants expressed that there is often a tendency for staff who lack appropriate digital literacy skills to rely on other staff to perform digital tasks.

**Table 2.** Examples of competencies being addressed elsewhere.

Domain	Competency	Area where participants identified competency has previously been addressed.
Domain 1: General	I have knowledge of and the ability to demonstrate foundational computer skills	Basic IT skills such as using email and Microsoft Word are often demonstrated when applying for an AHP role. Basic IT skills are required for AHPs to fulfil their role so are also often covered during the induction process.
Domain 2: Data Management and Clinical Informatics	I have knowledge and understanding of data management, information governance, and the risks associated with data privacy.	Processes related to data management and information governance are usually addressed within mandatory training modules.
Domain 2: Data Management and Clinical Informatics	I have the ability to recognise and use systems incorporating multi-factor authentication for transfer of patient data	Data security systems such as multi-factor authentication are usually addressed on an organisational level by the Trust IT department.
Domain 3: Records, assessments and plans	I have the ability to communication within own organisations to configure the rights and access to patient data held in the HER relative to the needs of self and others	Configuration rights and access to patient data is often governed by the Trust IT department and beyond the scope of an AHP.
Domain 4: Transfer of Care	I have knowledge and understanding of digital tools that support the transfer of patient information between health and care professionals at point of referral, admission handover or discharge	The ability to transfer patient data is required for AHPs to fulfil their role so is often addressed during the induction period.
Domain 5: Medicines management and optimisation	I have knowledge of and ability to identify appropriate rights and access required for independent prescribing	Prescribing medication often requires a separate qualification and is governed by a professional regulatory body.
Domain 6: Orders and results management	I have knowledge and understanding of digital tools which support visibility, requesting and resulting of medical testing.	Ordering and managing results is required for AHPs to fulfil their role so is usually addresses during the induction period.
Domain 7a: Assets and resource optimisation: Business related	I have knowledge and understanding of digital tools used locally for procurement and supply chain managements of goods and services	Business-related asset and resource optimisation is more relevant for staff in management roles.
Domain 7b: Assets and resource optimisation: Personal	I have the ability to access and manage own personal information via the ESR system	Personal asset and resources optimisation is usually addresses during the recruitment process or covered in HR induction.
Domain 8: Decision support	I have knowledge and understanding of the development and/or evaluation of clinical decision support systems which utilise machine learning and AI	Many processes related to decision making are beyond the scope of practice for an AHP; very few opportunities are available for AHPs to get involved in the development and/or evaluation of digital tools.
Domain 9: Digital therapeutics	I have the ability for self (or in the teaching of others) to use an online patient appointment booking system to reschedule, book or cancel and appointment	Processes related to managing patient appointments are more relevant to administrative roles.
Domain 10: Meta-competencies	I have the capacity to build relationships with key stakeholders for digital transformation within the local organisation or surrounding digital health ecosystem.	Meta-competencies are more relevant to staff in managerial roles.

*I see certain people in the department cover technology-wise for others … there's a lack of access to upskilling those people without it just coming from somebody who's already able to do it.* (Participant 1)

Participants highlighted that it is often assumed that staff have adequate levels of digital literacy and agreed that it would be helpful to have a baseline level of competency included in the AHP Digital Competency Framework, to ensure that everybody up to a similar standard.

*I think it's really important to recognise that not everybody is as IT savvy as some other people. It's sometimes taken for granted that you are more technologically minded than you might be.* (Participant 5)

*I think having that baseline, or that everyone's up to a core standard on qualifying in their role, would be really good*. (Participant 10)

In addition to the core competencies, participants explained that there should be additional competencies specific to different job roles that would become increasingly relevant as staff progress through their career. They suggested presenting the competencies as a pyramid to demonstrate the idea of building on previous knowledge and digital skills as they advance through their career. The discussion did not include how the competency framework could be effectively disseminated and integrated into clinical practice but rather focused on improving the framework and making competencies role specific.

*It almost needs to be specific to your trust, and the applications that you would be expected to use within your job role, which again might come back to the Banding or even just the particular area that you work in and what's required of you.* (Participant 6)

*I feel like you need it more like a pyramid visual and you could have the ones that are basic at the bottom, and then you could have arrows as you get to the top that go to other pages when you get to the point of taking about 8As, 8Bs, Band 9s etcetera … you could display it like that so that it's what it builds on.* (Participant 1)

Having identified several limitations of the AHP Digital Competency Framework, participants shared and discussed ideas about how the document could be further developed and improved. Recommendations for improvement can be found in Table 3.

**Table 3.** Recommendations to improve the AHP Digital Competency Framework.

Limitations	Recommendations
Use of ‘I have’ statements prompt a ‘yes/no’ answer without the requirement to demonstrate how the competency has been met.	Include a task-based element for each competency so staff can demonstrate their understanding of specific digital platforms or processes.
Competency statements too long and wordy.	Modify the competency statements to be shorter and phased using accessible language.
Competency statements too vague.	Include examples of digital platforms and processes specific to AHP practice.
Some competencies not relevant to all AHP roles and grades.	Re-structure the framework to include core competencies that are essential for all staff, and additional competencies that relate to specific job roles or Bands.

## Discussion

### Summary of findings

The purpose of the current study was to explore the opinions of AHPs towards the AHP Digital Competency Framework, through the use of focus groups. Participants acknowledged that digital technology is becoming increasingly more integrated within health and social care services and recognised the importance of AHPs having high levels of digital competency. There was consensus among participants that digital competencies should be assessed. Despite this, the majority of participants were unaware of the AHP Digital Competency Framework prior to taking part in the current study, suggesting that the dissemination and use of the framework within AHP practice is currently limited. The framework was developed by Health Education England as information and technology was one of the four identified AHP priorities. A panel of 40 AHPs reviewed several rounds of competencies resulting in 124 competencies within 10 domains and these domains were delineated by profession and band requirement although no detailed discrimination process was published with the framework.^8^

The use of technology in dietetic practice is embedded in the competency expectations for dietitians in the UK^13^ and has been highlighted as a priority for the profession.^14^ In addition, an overview of how digital technology is disrupting traditional dietetic practice underlined the need for dietitians to stay abreast of these advances and build their skills to improve their practice.^15^ A digital competency framework has also been developed for the pharmacy profession and research has raised similar concerns about lack of formal education in practice.^2^ Concerns about sufficient competence and a lack of skills were reported in AHPs in Sweden and Finland.^16^

The AHPs agreed that digital competencies should be assessed within the workplace, in order to ensure that staffs have the appropriate digital skills and capabilities to fulfil their role and confirms the findings from others.^2,7,16^ Peer assessment or assessment by senior staff members using the AHP Digital Competency Framework was highlighted as being potentially beneficial in helping AHPs to recognise areas that they find challenging and identify areas from improvement. Nevertheless, participants felt that the AHP Digital Competency Framework may not be appropriate to use as an assessment tool due to the lack of requirement to provide evidence that the competency had been met. For example, many of the competency statements began with the phrase ‘I have’, encouraging AHPs to merely response ‘yes’ or ‘no’ without having to actively demonstrate their capabilities. Interestingly, in a survey among 851 UK AHPs about digital competency, most respondents reported moderate–high self-rated confidence in using digital technologies for data management and clinical informatics but lower rates of confidence for the decision support and meta competency and records, assessments and plans domains. Overall, respondents reported high levels of motivation to develop their skills and a clear need to develop digital leadership and strategy development skills.^9^

Overall in this study, acceptance of eHealth and digital technologies was good whilst acknowledging that implementing changes into practice had various barriers. The participants commented on the AHP Digital Competency Framework structure and the content of the digital competency statements, expressing that some of the competencies were too vague and lacking in context. They discussed that this made it difficult to understand what technologies or processes were being referred to. Difficulties understanding the competencies were further exacerbated by the long and wordy nature of the competency statements. Participants also noted that not all of the competencies included in the AHP Digital Competency Framework were relevant to the AHP role, with some competency domains such as *Assets and resources optimisation: Business related* and *Meta-competencies* being more appropriate for staff at a managerial level. This was borne out by others,^16,17^ but unlike other studies, there was a high level of acceptance of the assessing and using the competency framework and participants recognising the need for tailored eHealth education.^18^ A recent publication examined the relative importance of 24 digital health technologies attributes among stakeholder groups and the most important attribute was ‘*Helps health professionals respond quickly when changes in patient care are needed*.’^19^ Given this finding was from a survey that included patients and carers, and community members as well as healthcare professionals, it clearly emphasises the importance of competent AHPs in relation to digital technologies. Technology-supported delivery modalities are in use in dietetic services and within dietetics, digital technology is used for nutrition assessment, diagnosis, intervention, and monitoring and evaluation which requires AHPs to be competent so that optimal care can be delivered.^15^ There is a clear need for a digitally competent workforce and advancing technology is seen as a key to transforming healthcare delivery.^6^ In terms of professional development, AHPs recognise the importance of being digitally competent but a clear plan of implementing the framework into practice is required. Using the framework across the AHP workforce has the potential to accurately measure digital skills and competencies and could be used in planning digital workforce programmes.^9^ Furthermore, there is a need for digital frameworks to be dynamic and consider the future requirements of the role so this there needs to be robust processes for ongoing development, dissemination and embedding in AHP practice.^20^

### Strengths and limitations

To the best of our knowledge, this is the first qualitative review of the AHP Digital Competency Framework in the UK and allowed the researchers the ability to undertake thematic analysis with the participants also providing feedback on how the framework could be improved. A limitation of the current study is the lack of variation in the AHP roles represented in the sample; with dietitians being the dominant profession. It may be that if more focus groups had been undertaken with other professions, the results may have differed. However, with an evaluation of pharmacists, similar results were observed.^2^ The focus groups were performed in London and the results may not represent other locations such as rural or remote locations. However, as the framework was developed for AHPs working the UK, we are confident that the findings could be applicable across the NHS.

## Conclusion

Overall, the AHP Digital Competency Framework has the potential to become a useful tool to support the enhancement of digital competency in AHPs and improve the delivery of patient care. However, there clearly is a need to increase awareness of the framework and greater dissemination. Improvements are needed in relation to methods of assessment, and the structure and content of the competency statements, for the framework to be used frequently and effectively in clinical practice.
